# Type I Interferon Signature in Chilblain-Like Lesions Associated with the COVID-19 Pandemic

**DOI:** 10.3390/dermatopathology7030010

**Published:** 2020-12-04

**Authors:** Roland Aschoff, Nick Zimmermann, Stefan Beissert, Claudia Günther

**Affiliations:** Department of Dermatology, Medical Faculty Carl Gustav Carus, TU Dresden, 01307 Dresden, Germany; roland.aschoff@ukdd.de (R.A.); Nick.zimmermann@ukdd.de (N.Z.); stefan.beissert@ukdd.de (S.B.)

**Keywords:** SARS-CoV-2, chilblain, type I interferon

## Abstract

Contemporarily to the new SARS-CoV-2 mediated COVID-19 pandemic, a rise in patients with acral chilblain lesions has been described. They manifest late after mild disease or asymptomatic exposure to SARS-CoV-2. Their pathogenic evolution is currently unknown. In biopsies from three patients with acral partially ulcerating chilblain lesions that occurred associated to the COVID-19 pandemic, we analysed the expression of type I interferon induced proteins and signal transduction kinases. Histology demonstrated perivascular and periadnexal lymphohistiocytic infiltrates and endothelial dominated MxA-staining, as well as pJAK1 activation. Our findings demonstrate induction of the type I IFN pathway in lesional sections of COVID-19-associated chilblain-like lesions. This may indicate a local antiviral immune activation status associated with preceding exposure to SARS-CoV-2.

## 1. Introduction

The pandemic of infection with the severe acute respiratory syndrome coronavirus (SARS-CoV-2) causing the atypical pneumonia coronavirus disease 19 (COVID-19) is ongoing [1]. It has become evident that the disease can affect not only the lungs but may proceed to a multisystemic disorder. Various skin manifestations have been observed, ranging from urticarial exanthema to Kawasaki syndrome. Concomitantly to the pandemic, several reports have described an increased incidence of ulcerative chilblain-like lesions that predominantly occur on the hands and feet [2]. The latter usually manifest several weeks after viral exposure and have been designated as “COVID toes” [3,4,5,6,7]. The pathophysiological correlation between chilblain-like lesions and exposure to SARS-CoV-2 was concluded from the increased incidence of the lesions in the context of the pandemic and their occurrence in persons who did not belong to a risk group of chilblain lesions or classical chilblain lupus, such as young men. It turned out that the connection was difficult to prove, because in most cases no positive nasopharyngeal swabs or autoantibodies could be detected [3,8].

Assuming that an effective antiviral response driven by type I interferon (IFN) production led to early control of the virus and might have prevented respiratory disease as well as antibody production, we aimed to examine histological sections of chilblain-like lesions for activation of the interferon system. Type I IFN cannot easily be detected due to its short half-life, therefore we labelled the antiviral protein myxovirus resistance protein A and analysed the activation of the IFN receptor. Stimulation of the type I or type II IFN receptor by IFNs leads to phosphorylation of the janus kinase (JAK)1 and JAK2 [9].

## 2. Patients and Methods

### 2.1. Patients

**Figure 1:**
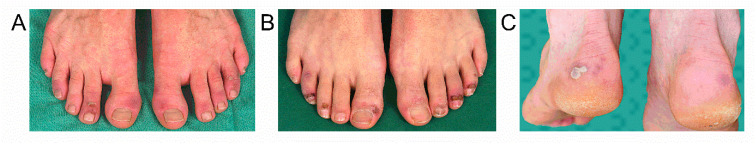
Clinical presentation of chilblain-like lesions on the feet and toes of three young men. Purple to red, tender chilblain-like lesions, superficial blisters and ulcerations on acral locations of the feet occurring 5 weeks after a travel through Germany in February 2020 in Patient 1 (**A**), 1 month after severe respiratory infection in Patient 2 (**B**), and 1 month after holidays in Tirol, Austria, that became a hotspot for COVID-19, in Patient 3 (**C**).

Here, we report three young Caucasian men with tender erythematous to purple partially ulcerative chilblain lesions on their feet and toes. Patient 1 was a 19-year-old man who spent holidays in a large city in northern Germany in February 2020. He experienced the first tender erythematous macules on his toes at the beginning of April (Figure 1A). Nasopharyngeal swabs and serology (anti-immunoglobin (Ig) A, IgG) for SARS-CoV-2 were performed at the end of April and showed negative results. Antinuclear antibodies were positive (1:320) without relevant subtyping. The patient was otherwise healthy and had no relevant medical history. Patient 2 was a 14-year-old boy who suffered from severe respiratory upper airway infection that affected the whole family in February 2020 during the beginning of COVID-19 epidemic in Germany. He detected first tender red macules, papules, and ulcerations on his toes at the beginning of April (Figure 1B). Nasopharyngeal swabs and serology (anti-IgA, IgG) for SARS-CoV-2, as well as PCR testing for SARS-Cov-2 E gen in lesional skin obtained from the feet were negative in May. Patient 3 was a 26-year-old otherwise healthy young man who experienced the first lesions on his feet in mid-March after spending ski holidays in the Tirolian Alps in mid-February 2020 (Figure 1C). He did not report respiratory infection. Pharyngeal swabs, serologic testing, and PCR testing for SARS-Cov-2 E gen in lesional skin remained negative in April. Biopsies were performed in all three individuals at the time of their presentation (end of April in Patients 1 and 3, beginning of May in Patient 2). In all patients, chilblain-like lesions resolved with topical mometasonfuroate treatment after 2–3 months. All patients gave their written informed consent for publication. The study was approved by the ethics committee of the Medical faculty of TU Dresden (EK169052010).

### 2.2. Immunohistochemistry

Biopsies were deparaffinised and antigen retrieval was performed (CC1 (#950-124 Ventana Medical Systems Inc., Oro Valley, AZ, USA), 60 min). Thereafter, skin sections were incubated with mouse IgG-anti-myxovirus resistance protein A-antibody (MxA, O. Haller, Freiburg, 1:600), or anti-phosphoJAK (pJAK)1-antibody, 1:200, abcam, #ab138005 or anti-pJAK2-antibody, 1:200, abcam, #ab32101 diluted in PBS with 1% FCS for 30 min at 37 °C using BenchMark XT (Ventana). The alkaline phosphatase detection kit with FAST red was used to visualize the staining (#760-501, Ventana). Direct immunofluorescence was performed on cryostat sections using anti-human complement C3c-Fitc (DakoCytomation #F0201), anti-human IgG-Fitc (DakoCytomation # F0317) and anti-human IgM-Fitc (DakoCytomation # F0315). Slides were incubated with antibodies for 30 min at room temperature, mounted in Vectashield mounting medium (Vector Labs, Burlingame, CA, USA) and analysed with a Zeiss Axio Imager A1 microscope.

## 3. Results

**Figure 2:**
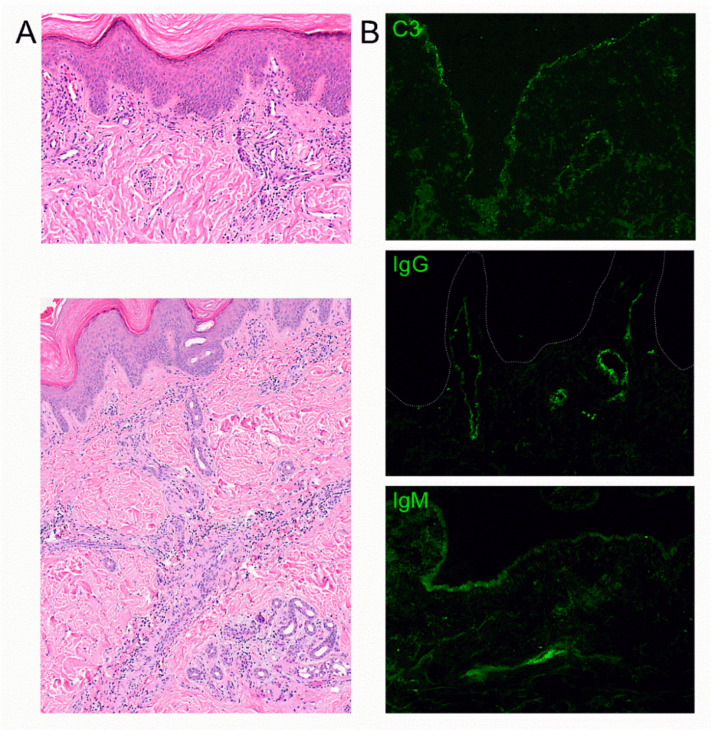
Histological pattern of chilblain-like lesions. Hematoxylin Eosin (HE) staining of chilblain-like lesions demonstrating superficial and deep perivascular and periadnexal lymphocytic infiltrates (**A**), magnification ×200 and ×100. Immunofluorescence staining shows deposition of c3, IgG and IgM in dermal vessels and granular c3 and IgM deposits at the basement membrane zone (**B**), magnification ×400. Histology of Patient 2 is shown as representative of similar results in all three patients.

**Figure 3:**
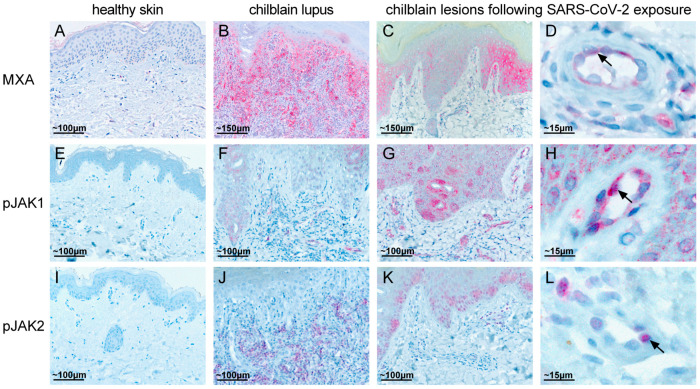
Immunohistochemistry demonstrating type I IFN activation. (**A**–**D**): Staining for MxA in red is negative in healthy skin (**A**), shows strong staining in conventional chilblain lupus (**B**), and protein induction in epidermis, dermis and endothelial cells (arrow) in lesional skin of chilblain-like lesions after exposure to SARS-CoV-2 (**C**,**D** arrow). (**E**–**H**): Staining for pJAK1 in red is negative in healthy skin (**E**), shows faint staining in conventional chilblain lupus (**F**), and staining in the epidermis, dermis and endothelial cells (arrow) in lesional skin of chilblain-like lesions after exposure to SARS-CoV-2 (**G**,**H**). (**I**–**L**): Staining for pJAK2 in red is negative in healthy skin (**I**), shows staining in chilblain lupus (**J**), and faint staining in the epidermis, dermis and endothelial cells (arrow) in lesional skin of chilblain-like lesions after exposure to SARS-CoV-2 (**K**,**L**). (**C**,**D**,**G**,**H**,**K**,**L**) show representative images of stainings performed in all 3 patients. D, H, L show magnifications of the respective staining in (**C**,**G**,**K**).

We observed a perivascular and periadnexal lymphocytic infiltrate extending to the mid and lower dermis in all samples. There were small, dilated vessels in the upper dermis but no significant interface dermatitis (Figure 2A). Mucin deposition was detectable in the dermis in alcian blue-stained sections. Immunofluorescence staining demonstrated the deposition of C3, IgG and IgM in small vessels and partial granular deposits at the basement membrane zone (Figure 2B). Immunohistochemistry revealed positive staining for MxA in the upper dermis and epidermis (Figure 3C,D) that was not detected in healthy skin (Figure 3A). MxA was detected in endothelial cells of most vessels (Figure 3C,D). The staining was comparable to MxA staining detected in chilblain lupus (Figure 3B). Type I IFN activation was further suggested by positive staining for pJAK1 that is involved in signal transduction of the type I IFN receptor (Figure 3E–H) [9]. pJAK1 was mainly expressed in the endothelia and epithelium of conventional chilblain lupus as positive control (Figure 3F), as well as chilblain-like lesions observed during the COVID-19 pandemic (Figure 3G,H). The staining was negative in healthy skin (Figure 3E). Staining for pJAK2 was weaker and located to vessels, epidermis and few inflammatory cells in chilblain-like lesions (Figure 3K,L). It was also expressed in chilblain lupus (Figure 3J). The staining for pJAK2 was negative in healthy skin (Figure 3I). JAK2 is phosphorylated upon ligation of the type II IFN receptor that is activated by interferon gamma [9].

## 4. Discussion

Here, we provide evidence for a role of type I IFN in the pathogenesis of chilblain-like lesions observed during the COVID-19 pandemic. Staining for MxA revealed positive deposition of this type I IFN induced protein in the endothelial cells and surrounding tissue. Lesional endothelial cells also expressed pJAK1 indicating an activation of the type I IFN receptor that signals via JAK1 and TYK2 [9].

A direct viral infection of endothelial cells by SARS-CoV-2 was demonstrated in post-mortem histological sections of patients with severe cardiovascular complications during COVID-19 [10,11,12]. In addition, staining of the SARS-CoV-2 spike protein in endothelial cells of chilblain-like lesions has been demonstrated [12]. This direct infection of endothelial cells may be sufficient to induce local type I IFN induction and chilblain-like lesions. In our cases, RT-PCR testing of SARS-CoV-2 in lesional tissue remained negative. It was also negative in other reports of chilblain lesions concomitant to the COVID-19 pandemic [13]. The PCR test in tissue samples might not be sensitive enough if infection occurs only in endothelial cells.

The role of the type I IFN pathway in the induction of chilblain-like lesions is further supported by the observation that patients with chronic type I IFN activation due to rare type I interferonopathies frequently develop chilblain lesions [14,15,16].

Histologically, we did not observe an interphase dermatitis that can be seen in certain stages of chilblain lupus. Acute chilblain lupus usually presents with interphase dermatitis, but later stages might only show upper and deep perivascular and periadnexal infiltrates. This observation has been reported by others investigating chilblain-like lesions during the pandemic [6,17]. One reason might be that the biopsy was taken in a late disease stage.

Patients with chilblain-like lesions during the COVID-19 pandemic mostly did not report severe COVID-19 disease before onset of the lesions, but only respiratory symptoms that usually did not require admission to the hospital. Often, the chilblain-like lesions occurred with a long delay up to 30 days after infection. The mild symptoms and the delayed onset might be the reason for the low positive rate of nasopharyngeal swabs and the lack of an antibody response determined in these patients [3]. The viral infection might have been controlled very fast and efficiently by innate immune system via the induction of type I IFN [18]. This has been described to protect from severe respiratory disease manifestation [19]. The activated IFN response in patients with chilblain lesions after SARS-CoV-2 exposure has also been suggested to explain the repression of autoantibody formation due to a type I IFN driven deletion of antiviral B cells in the beginning of an infection [17,20].

However, due to the lack of positive tests for SARS-CoV-2 by RT PCR following nasopharyngeal swab or serologic testing for SARS-CoV-2-specific IgM or IgG antibodies, other authors questioned the association of acral chilblain-like lesions and SARS-CoV-2 exposure [13,21,22]. These authors proposed that lifestyle changes imposed by the quarantine, such as walking barefoot in unheated homes, inactivity, and time spent in sedentary positions, could explain these findings [22]. In our cases, we rather exclude those possibilities due to the warm season, the normal body mass index and less restrictive quarantine in the area. The patients were always allowed to continue their outdoor activities.

Further detailed observation of the course of infection with SARS-Cov-2 and larger case series as well as detailed immunological analysis are necessary to prove the potential relationship of chilblains to SARS-Cov-2 exposure which may also help to understand pathogenesis of chilblain lesions in general.

## 5. Conclusions

We observed an activation of the type I IFN immune response in lesional tissue of chilblain-like lesions occurring during the COVID-19 pandemic. Endothelial cells expressed the type I IFN induced protein MxA and pJAK1 indicating type I IFN receptor activation. This was associated with complement and Ig deposition in lesional vessels. According to the current state of knowledge this may indicate that chilblain-like lesion are induced by a local type I IFN driven immune response to endothelial infection with SARS-CoV-2 in patients without severe COVID-19 organ manifestations.
